# Determination of lower radiation dose limit for automatic measurement of adipose tissue

**DOI:** 10.1002/acm2.13958

**Published:** 2023-04-06

**Authors:** Andrew T. Grainger, Akira Hasegawa, Arun Krishnaraj

**Affiliations:** ^1^ Radiology & Medical Imaging School of Medicine University of Virginia Charlottesville Virginia USA; ^2^ Department of Radiological Technology National Cancer Center Japan Tokyo Japan; ^3^ AlgoMedica, Inc. Sunnyvale California USA

**Keywords:** AI, CT, fat, low‐dose, quantification

## Abstract

The purpose of this study was to determine the lower limit of radiation dose required to measure visceral adipose tissue (VAT) and subcutaneous adipose tissue (SAT) volumes when a fat quantification and noise reduction techniques (NRTs) are combined. For this purpose, we utilized CT colonography (CTC) images taken at low doses and manually segmented VAT and SAT fat volumes as ground truth. In order to derive the acceptable precision of the measurements needed to estimate the lower limit of radiation dose, we estimated the effect of different positioning during CT scanning on fat measurements using manually segmented VAT and SAT against normal dose. As a result, the acceptable accuracy of SAT and VAT was found to be 94.5% and 85.2%, respectively. Using these thresholds, the lower radiation dose limit required to accurately measure SAT using 5.25‐mm slice‐thick images was 1.5 mGy of size‐specific dose estimates (SSDE), while the lower radiation dose limit required to accurately measure VAT was 0.4 mGy of SSDE. The lower dose limit for SAT and VAT combined was 1.5 mGy, which was equivalent to an estimated effective dose of 0.38 mSv. Alternatively, without noise reduction, SAT could not achieve acceptable accuracy even for images with a slice thickness of 5.25 mm, while VAT required noise reduction for images with a slice thickness of 1.25 mm, but could achieve acceptable accuracy without noise reduction for images with a slice thickness of 5.25 mm.

## 1 INTRODUCTION

Obesity, which refers to the accumulation of excess fat in the body, is a contributing factor in many disease processes worldwide and has a disproportionate impact on the U.S. population, which has experienced a marked increase in obesity levels over the last 50 years.^1^, ^2^ Historically, body mass index (BMI), waist circumference, and waist‐to‐hip ratio have been used to diagnose obesity by indirectly predicting body fat. However, although these indices are strongly correlated with body fat percentage, the predictive ability to estimate an individual's body fat is limited as the weight of skeletal muscle is not accounted for and direct assessment of fat deposition is not obtained.

Negative cardiovascular disease outcomes are more closely associated with high visceral adiposity than with total body fat.^3^, ^4^ Moreover, there are considerable individual differences in the amount of subcutaneous and intra‐abdominal adipose tissue in the abdominal cavity, regardless of BMI or level of total adiposity. This observation has led to growing interest in using diagnostic imaging techniques such as CT imaging to directly measure and monitor different fat deposits: visceral adipose tissue (VAT) and subcutaneous adipose tissue (SAT).^5^, ^6^, ^7^

There are, however, two limitations to routinely obtaining CT scans to measure and monitor fat: first, the effort and time required to quantify SAT and VAT, and second, patient radiation exposure. To solve the first problem, many semi‐automatic and fully automatic methods have been developed. In the early 2010s, Kim et al. reported a fat quantification technique algorithm based on a separation mask algorithm,^8^ and Nemoto et al. reported a fully automatic fat quantification technique using image segmentation.^9^ Beginning around 2020, numerous studies have leveraged deep learning to perform automatic fat quantification.^10^, ^11^, ^12^, ^13^, ^14^, ^15^, ^16^, ^17^, ^18^, ^19^, ^20^

Regarding the second limitation, patient radiation exposure, the guiding principle of medical imaging has been “as low as reasonably achievable” (ALARA). Yamada et al. reported the lower limit of radiation dose of CT images for manual fat quantification.^21^ However, to the best of our knowledge, no study has reported the lower limit of radiation dose for automated fat quantification. Many of the reports on automated fat quantification methods provide insufficient information on radiation dose or use doses used for routine abdominal scans.^11^, ^12^, ^13^, ^14^, ^16^, ^17^, ^18^, ^19^, ^20^ Since the early 2010′s, Pickhardt et al. have published a series of reports on the application of fat quantification to abdominal CT for the purpose of colorectal cancer prevention and screening, using the CT colonoscopy (CTC) technique.^10^, ^12^, ^22^, ^23^ Performing opportunistic fat quantification screening on CTC images is one method of obtaining fat quantification information without additional radiation exposure. However, the volume of CTC is still relatively limited, so effective opportunities to screen for metabolic co‐morbidities such as high VAT on healthy individuals would require purposeful low‐dose CT imaging. For this reason, it is important to investigate the lower limit of radiation dose for fat quantification.

In recent years, various vendors have offered a wide range of noise reduction techniques (NRTs) based on iterative reconstruction,^24^, ^25^, ^26^ deep learning, and other methods.^27^, ^28^, ^29^ The limitation of these NRTs is that noise reduction is generally performed during image reconstruction, so these methods cannot process images that have already been reconstructed or images taken with a different scanner. An alternative approach is to perform noise reduction as post‐processing on reconstructed images. This technique is scanner agnostic and has the advantage of being applicable to prior images stored in PACS.^30^, ^31^, ^32^

It is reasonable to assume that noise due to the lower radiation dose could be the cause of the loss of accuracy in fat quantification when radiation dose is markedly reduced. Hence, NRTs are likely to be useful in lowering the lower limit of radiation dose required for fat quantification. However, Yamada et al. reported that NRTs did not contribute to further dose reduction during manual fat quantification.^21^ It remains to be determined if this is true for lower doses than those reported, especially for our automated fat quantification.

The purpose of this study is to investigate the lower limit of radiation dose required for accurate fat quantification when using automated fat quantification. We attempted to determine the lower limit of the required radiation dose using a combination of our own deep learning‐based fat quantification technique and a vendor‐agnostic NRT, PixelShine (AlgoMedica, Inc., Sunnyvale, CA),^27^, ^30^, ^33^ and also evaluated the contribution of the NRT to radiation dose reduction. For this purpose, we used CT images obtained at our institution from CT colorectal screening examinations and standard dose CT images taken within 6 months before and after the examination. Using the values obtained from the manually segmented VAT and SAT of these images, we estimated the effect of different positioning during CT scanning on fat quantification, which was defined as the acceptable accuracy required for fat quantification techniques. Furthermore, the accuracy of fat quantification was evaluated using size‐specific dose estimates (SSDE), considering that image noise is highly dependent on patient size as well as dose.

## 2 METHODS

In order to investigate the lower radiation dose limit at which fat quantification can be performed, CTC images taken at low doses were collected. A cohort of adult CTC cases with an extracolonic finding rating of E3^34^ or higher were selected from CTC images taken between November 2019 and December 2020, having images of standard‐dose CT scans taken for follow‐up or another purpose within 6 months from the CTC examination. There were 48 patients who fell into this category. Of those 48 patients, one patient was excluded because of a body size difference between standard dose and CTC images that raised suspicion of a different patient, and another was excluded because of severe metal artifacts from metallic spinal implants. The rest of the 46 patients included 23 males and 23 females, and the age range of the patients was 35 to 74. The acquisition and reconstruction parameters of the CTC are shown in Table 1. As shown in this table, a fixed mA has been used for the CTC examinations with a CTDIvol of 1.7 mGy independent of this study. Images reconstructed with a slice thickness of 1.25 mm were used for CTC, but additional images with a slice thickness of 5.25 mm were also reconstructed for this study.

**TABLE 1:** Acquisition and reconstruction parameters for CT colonography (CTC).

Manufacturer	GE
Manufacturer model name	Discovery CT750 HD
Scan options	HELICAL MODE
Revolution time	0.5 s
Total collimation width	20 mm
Single collimation width	0.625 mm
Spiral pitch factor	1.375
Table feed per rotation	27.5 mm/rot
KVP	120 kV
X‐ray tube current	60 mA
Effective mAs	21.82 mAs
Filter type	BODY FILTER
Data collection diameter	500 mm
CTDIvol	1.7 mGy (Body 32)
Convolution kernel	SOFT
Matrix size	512 x 512 pixels
Reconstruction diameter	depends on case
Thickness	5.25 mm
Slice interval	5 mm

Alternatively, since images of the normal radiation doses were taken with different parameters, the details of the acquisition parameters are not described. But the CTDIvol used ranged from 5.5 to 31.4 mGy (mean 16.9 mGy with a standard deviation of 5.5 mGy).

Noise reduction was performed using PixelShine's US (Ultra‐low dose soft tissue) mode on all 46 CTC images with slice thicknesses of 1.25 and 5.25 mm. From the images with and without noise reduction, slices corresponding to the T12‐L5 vertebral body locations were extracted and fat segmentation was performed to quantify SAT and VAT, as previously reported.^20^

When quantifying SAT and VAT, fat volume $V_{t}$ is usually calculated by the following Equation (1):

(1) $$V_{t}=\sum_{i=1}^{N}S_{i}\cdot \;I$$

where $S_{i}$ is the area of fat in the *i*‐th slice, *N* is the total number of slices, and *I* is the slice interval. However, the scan length from T12 to L5 cannot be precisely determined, and there is a large variation among scans and reconstructed images. There are also variations in slice thickness and slice intervals depending on the reconstructed images. All of these variations have a significant impact on the accuracy of fat volume $V_{t}$. In order to minimize potential errors due to these factors, the fat volume $V_{u}$ per unit scan length was calculated and used instead of fat volume in this paper using the following Equation (2):

(2) $$V_{u}=\frac{V_{t}}{L}=\sum_{i=1}^{N}S_{i}\cdot I/\sum_{i=1}^{N}I=\frac{1}{N}\sum_{i=1}^{N}S_{i}$$

That is, it is the average value of the fat area calculated in each slice. In the following, fat volume per unit scan length will be referred to simply as fat volume.

### 2.1 Manual segmentation and ground truth

The 5.25 mm CTC images without noise reduction and the images taken at normal dose were manually segmented into SAT and VAT one by one by a radiologist with more than 20 years of experience. Using these segmented images, the fat volume of SAT and VAT were calculated, respectively, and the fat volume of SAT and VAT obtained from the normal dose images was used as ground truth.

### 2.2 Definition of measurement accuracy

For ground truths, the accuracy of the measured SAT and VAT was defined by the following Equation (3):

(3) $$Accuracy\;\%\;=\left(1.0-\frac{\left|V_{true}-V_{measure}\right|}{V_{true}}\right)\;\times 100$$

where $V_{true}$ is the volume of fat used as ground truth and $V_{measure}$ is the measured fat volume.

### 2.3 Estimation of allowable accuracy

When estimating the lower dose limit for SAT and VAT measurements, some tolerance is required. When measuring fat volume for SAT and VAT, the following factors may have an influence: (1) measurement error due to fat quantification techniques, (2) differences in parameters during image reconstruction such as reconstruction FOV, slice thickness, and slice spacing, (3) changes in body shape and fat composition, and (4) differences in positioning during CT imaging. Of these, (1)–(3) are avoidable while (4) is not. Therefore, it is practically meaningless to improve the measurement accuracy of fat measurement techniques to make the accuracy less than the error due to positioning. With these considerations in mind, we attempted to estimate the error due to differences in positioning during CT imaging, believing that the acceptable range of error is determined by the difference in positioning during CT imaging.

In order to eliminate the influence of measurement errors caused by fat quantification techniques, we used both CTC images and images taken at normal dose, and fat volumes of SAT and VAT calculated from manually segmented images were used for the evaluation described below. The use of CTC images and normal dose images may introduce errors due to differences in dose, but since fat quantification with CTC images has already been actively studied, we considered the errors due to differences in dose to be sufficiently small. The accuracy of SAT and VAT computed from manually segmented CTC images using SAT and VAT obtained from the ground truth images (manually segmented normal dose images) was then calculated and used to evaluate the following.

### 2.4 Effects of different reconstruction parameters

Among the reconstruction parameters that may affect fat volume, the effects of slice thickness and slice intervals can be minimized by using the fat volume per unit scan length in Equation (2) instead of the fat volume expressed in Equation (1). On the other hand, the effect of truncation of a body part due to reconstruction FOV can only be determined statistically from images in which truncation is actually occurring. Therefore, we visually identified cases in which at least one of the standard dose images or CTC images was truncated by FOV. As a result, 17 cases with truncation were identified. In two of these cases, truncation occurred in both the standard dose image and the CTC image. Next, to determine whether the presence or absence of truncation statistically affects the accuracy of SAT and VAT measurements, a two‐sided Mann–Whitney U test was conducted at the 5% significance level with the null hypothesis that the presence or absence of truncation does not affect the accuracy of SAT and VAT measurements individually. This allowed us to examine whether or not there was a need to eliminate the influence of differences in parameters during image reconstruction.

### 2.5 Effects of changes in body shape and fat composition

CTC images and normal‐dose images have different acquisition dates. Therefore, differences due to changes in body shape and fat composition may exist. Therefore, assuming that there is little change in body shape and adipose tissue within 2 weeks, we tested whether there is a significant difference in fat quantification between scans more than 2 weeks apart in the normal‐dose and CTC 5.25 mm images. To do so, a two‐sided Mann–Whitney U test at the 5% significance level was performed with the null hypothesis that SAT and VAT measurements are equivalent between scans less than 2 weeks and more than 2 weeks apart.

### 2.6 Estimation of impact of different positioning

After eliminating the effects of (1), (2), and (3) using the method described above, the effects of positioning differences on SAT and VAT were estimated. We calculated the average value of measurement accuracy using only data taken within the acquisition interval, which is considered to be less affected by changes in body shape. This average value was then defined as the acceptable margin of error for fat quantification.

### 2.7 Estimation of lower radiation dose limits

Using the obtained error tolerance, the accuracy of our fat quantification technique with and without noise reduction for CTC image data with a slice thickness of 5.25 mm was evaluated using Equation (3). Considering that image noise is highly dependent on patient size as well as dose, we plotted the accuracy of fat quantification against SSDE and compared it to the acceptable accuracy. Note that SSDE was calculated using the sum of anterior‐posterior (AP) and lateral (LAT) dimensions.^35^

To further examine the accuracy at even lower doses, we evaluated the accuracy of SAT and VAT using 1.25 mm slice thickness data of the same CTC case, as we did with 5.25 mm data. The noise level in CT images is inversely proportional to the square root of the radiation dose and inversely proportional to the square root of the slice thickness. Based on this, the noise level in a CT image with a 1.25‐mm slice thickness is considered to be almost the same as that of a 5.25‐mm slice thickness image acquired with a 1/4 ( = 1.25/5.25) dose, assuming that the other conditions under which the CT image is acquired are the same. Therefore, we defined the SSDE corresponding to a slice thickness of 5.25 mm as

(4) $$SSDE_{5.25}=\;SSDE\times \frac{Thickness}{5.25}$$

and the measurement accuracy of SAT and VAT in CTC images with a slice thickness of 1.25 mm is plotted against SSDE5.25, along with the acceptable accuracy. In this way, the results were predicted for SSDEs between 0.35 and 0.60 mGy.

In order to find the lower limit of dose at which the accuracy of SAT and VAT measurements is above acceptable accuracy, SSDE 5.25 was divided at 0.5 mGy intervals to test whether there is a difference between the accuracy of SAT and VAT measurements and error due to differences in positioning. A one‐sided Mann–Whitney U test was performed at the 5% level of significance.

## 3 RESULTS

### 3.1 Fat quantification in CT colonoscopy cases

**FIGURE 1:**
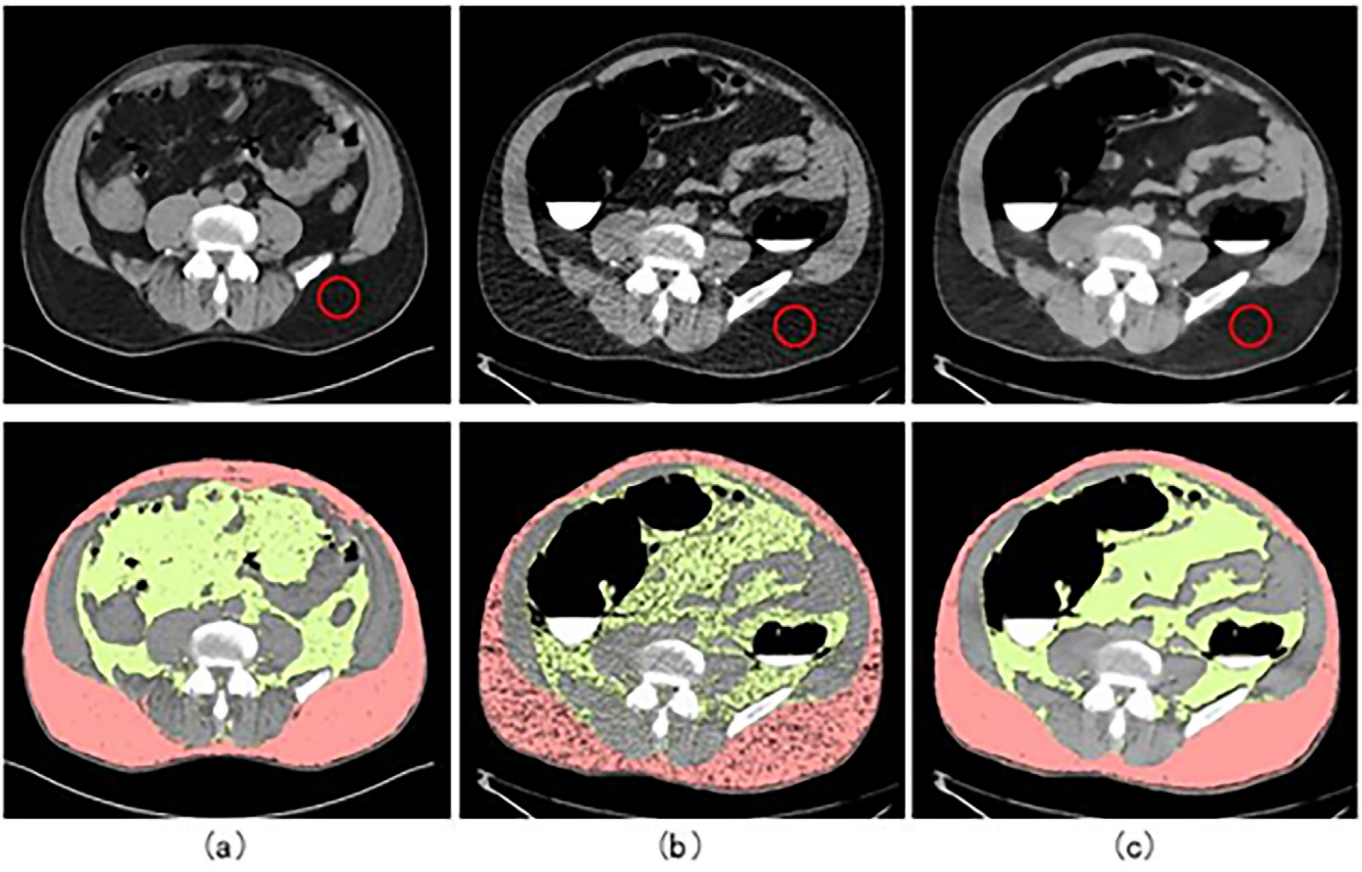
(a) An abdominal image (top) and fat segmentation results (bottom) taken at the standard dose of CTDIvol 18.0 mGy. (b) Same patient's CT colonography (CTC) image (top) acquired at CTDIvol 1.7 mGy within 6 months of the standard dose image and the fat segmentation result (bottom), and (c) the denoised image of the CTC image (top) and its fat segmentation result (bottom). The area used to determine noise is delineated by a red circle in each image (top).

Figure 1 shows a standard dose abdominal image, a CTC image of the same patient taken within 6 months, its denoised image, and the fat segmentation for each. In this case, the standard dose image was taken at CTDIvol 18.0 mGy (SSDE 20.4 mGy) and the CTC images were acquired at CTDIvol 1.7 mGy (SSDE 1.9 mGy). In this example, the low‐dose image was taken at only 10% of the standard dose image, resulting in a much noisier image than the standard dose one. After noise reduction, the noise in the image is improved to a level comparable to the standard dose. The noise (standard deviation) was measured in the fatty area at the lower right of each image and was on average12.7 HU for the standard dose image, 33.8 HU for the low‐dose image without noise reduction, and 11.0 HU for the image after noise reduction. As a result, for fat segmentation images, the results were clearly improved after noise reduction.

### 3.2 Effects of changes in body shape and fat composition

Table 2 shows the results of testing whether significant differences exist between standard dose images and CTC images with and without truncation by FOV. The fat volumes of SAT and VAT by manual segmentation were compared in 17 cases where at least one of the standard dose images or CTC images was truncated by FOV and in 29 cases where no truncation was performed. As shown in this table, *p* > 0.05 for both SAT and VAT, indicating that the presence or absence of truncation by FOV does not affect the measurement accuracy of SAT and VAT.

**TABLE 2:** Effect of truncation by reconstructed FOV on SAT and VAT measurement accuracy.

	Truncation			No truncation			*p*‐value
	Samples	Mean	SD	Samples	Mean	SD	
SAT	17	87.8%	9.8%	29	91.6%	7.0%	0.14
VAT	17	88.3%	9.4%	29	81.4%	15.1%	0.11

*Note*: In both cases, there was no significant difference in measurement accuracy with and without truncation.

Abbreviations: SAT, subcutaneous adipose tissue; VAT, visceral adipose tissue.

### 3.3 Effects of changes in body shape and fat composition

**FIGURE 2:**
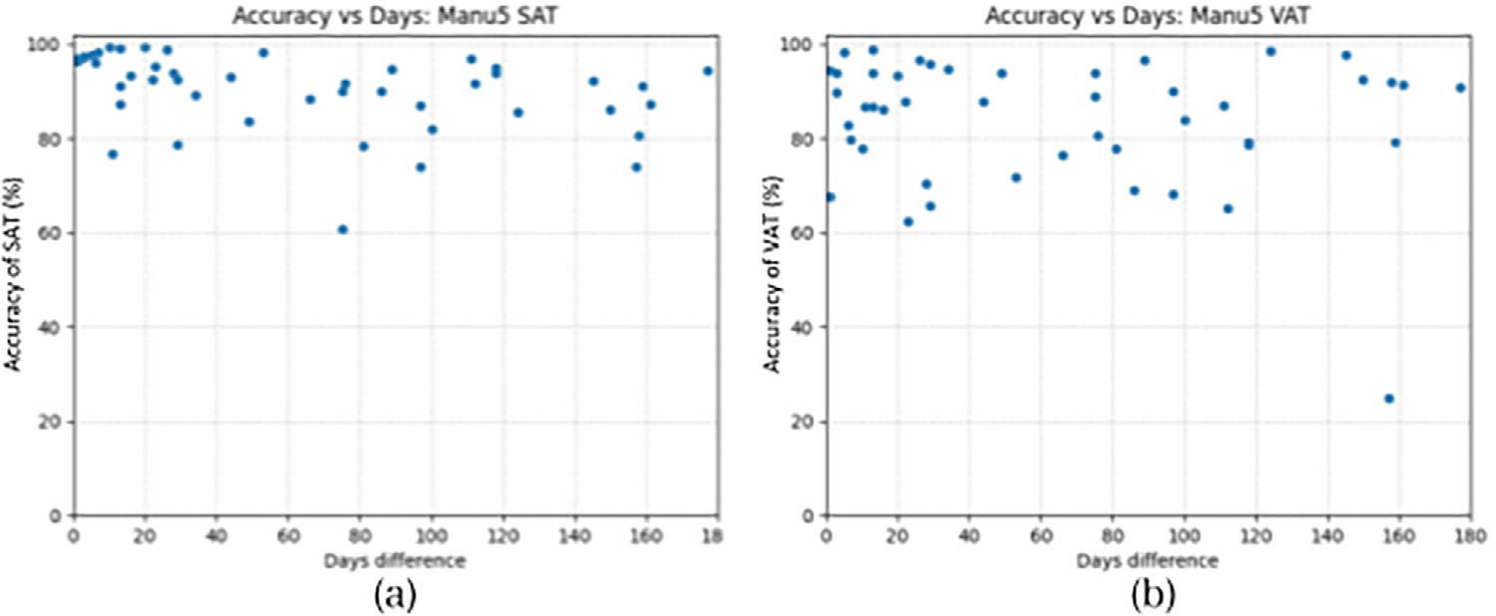
(a) Subcutaneous adipose tissue (SAT) and (b) visceral adipose tissue (VAT) measurement accuracy versus days between scans.

Figure 2 shows a plot of SAT and VAT measurement accuracy versus the number of days between imaging. Table 3 shows the results of testing the null hypothesis that SAT and VAT measurements are equivalent for manually segmented normal dose images and CTC 5.25 mm images with an acquisition interval of less than 2 weeks and more than 2 weeks. For SAT, there was a difference between values of less than 2 weeks and values of more than 2 weeks (*p* = 0.01). On the other hand, for VAT, there was no difference between values of less than 2 weeks and values of more than 2 weeks (*p* > 0.05).

**TABLE 3:** Effect of body shape changes due to differences in the number of days between scans on the accuracy of SAT and VAT measurements; significant differences were found for SAT, but not for VAT.

	Difference < 15 days			Difference > = 15 days			*p*‐value
	Samples	Mean	SD	Samples	Mean	SD	
SAT	12	94.5%	6.6%	34	88.7%	8.3%	0.01
VAT	12	87.6%	9.2%	34	82.7%	14.7%	0.36

Abbreviations: SAT, subcutaneous adipose tissue; VAT, visceral adipose tissue.

### 3.4 Estimation of impact of different positioning

Based on these results, the effects of positioning differences on SAT and VAT were estimated. For SAT, we calculated the average of measurement accuracy only for data taken within 2 weeks and found an average accuracy of 94.5%. On the other hand, for VAT, the average value of all data was calculated, excluding one case of obvious outliers (accuracy in the 20% range and more than 3σ from the average value) observed in Figure 2b. As a result, the average accuracy of VAT was 85.2%. From the above, it can be understood that as long as there are differences in positioning, the accuracy of SAT will remain around 94.5%, while the accuracy of VAT will reach only 85.2% on average.

### 3.5 Estimation of lower radiation dose limits

**FIGURE 3:**
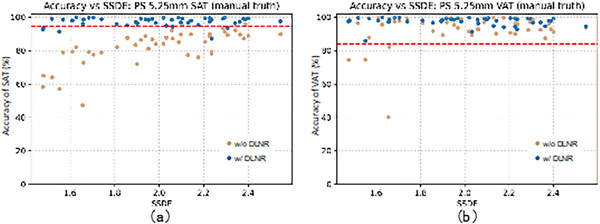
Using the manually calculated subcutaneous adipose tissue (SAT) and visceral adipose tissue (VAT) values for CT colonography (CTC) 5.25 mm slice thickness images as ground truth, the fat quantification accuracy before and after noise removal was plotted against size‐specific dose estimates (SSDE); (a) SAT, (b) VAT. In the figure, the red line is the mean measurement accuracy due to different positioning, which is considered to be the accuracy required for the fat quantification technique.

Based on the above, we evaluated the accuracy of our fat quantification technique with and without noise removal using the manually calculated SAT and VAT values for CTC images as ground truth. Figure 3 plots the relationship between SAT and VAT measurement accuracy and SSDE for images with a slice thickness of 5.25 mm, and the acceptable accuracy is indicated by the dotted line. Without noise reduction, the measurement accuracy of SAT was clearly below the acceptable threshold, but noise reduction improved it to almost exceed the acceptable threshold. On the other hand, for VAT without noise removal, the accuracy is above the threshold in most cases except in some cases with low SSDE. After noise reduction, all cases exceeded the threshold.

**FIGURE 4:**
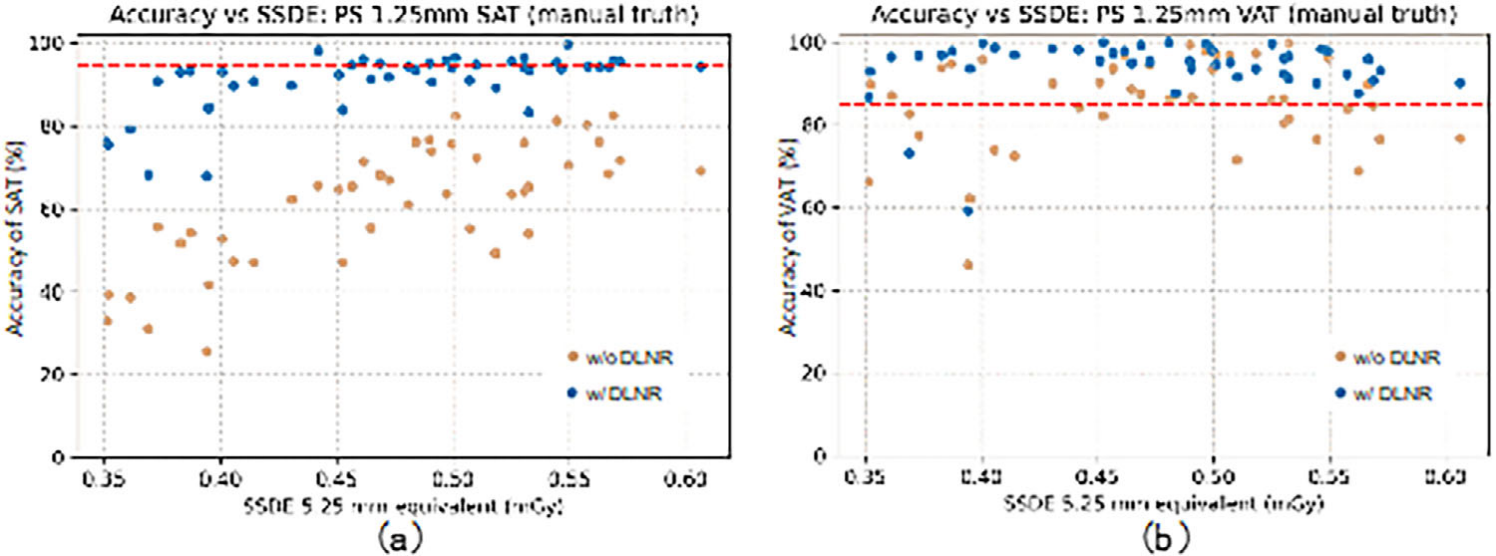
Using the manually calculated subcutaneous adipose tissue (SAT) and visceral adipose tissue (VAT) values for CT colonography (CTC) 5.25 mm slice thickness images as ground truth, the fat quantification accuracy before and after noise removal on CTC 1.25 mm slice thickness images was plotted against the 5.25 mm slice thickness equivalent size‐specific dose estimates (SSDE); (a) SAT, (b) VAT. In the figure, the red line indicates the average measurement accuracy due to different positioning.

In images with a slice thickness of 1.25 mm, for VAT without noise reduction, the accuracy was below the threshold in many cases (Figure 4). However, noise reduction improved the accuracy to above the threshold in most cases.

Tables 4 and 5 show the results of testing the accuracy with and without NRT for CTC images (5.25 and 1.25 mm), as well as the accuracy in each dose range with NRT versus the average accuracy when positioning differences are included (SAT 94.5%, VAT 85.2). Here, the SAT and VAT values calculated from manually segmented images on CTC 5.25 mm images were considered ground‐truth. For SAT without noise reduction, the results were almost always less than acceptable accuracy for both 5.25 and 1.25 mm slice thicknesses (*p* < 0.05) (Table 4). But for 5.25 mm, the noise reduction raised the measurements almost always above acceptable accuracy (*p* = 0.957). For the 1.25‐mm‐thick images, the *p*‐values were smaller than 0.05 for all intervals except for the 0.4–0.45 mGy range for SSDE_5.25_. In the 0.4–0.45 mGy range, *p* = 0.117, but the sample size was small. Considering these facts, the lower limit of SSDE_5.25_ required for SAT measurements could be considered to be between 0.5 and 1.5 mGy, but based on the present data alone, we can conclude that it is 1.5 mGy.

**TABLE 4:** Using the manually calculated SAT value for CT colonography (CTC) 5.25 mm images as the ground truth, the accuracy of SAT with and without NRT for CTC images (5.25 and 1.25 mm), as well as the accuracy of SAT in each dose range with NRT, was tested against the average accuracy of SAT with different positioning (94.5%).

	Samples	Mean	SD	*p*‐value
SAT manual (difference < 15 days)	**12**	**94.5%**	**6.6%**	–
Without NRT				
SAT ‐ 5.25 mm	46	82.2%	10.3%	<0.001
SAT ‐ 1.25 mm	46	61.3%	14.5%	<0.001
With NRT				
SAT ‐ 5.25 mm (SSDE_5.25_ ≥ 1.5)	46	97.4%	2.6%	0.957
SAT ‐ 1.25 mm	46	90.9%	7.2%	0.003
0.35 ⩽ SSDE_5.25_ < 0.4	9	80.9%	9.9%	0.001
0.4 ⩽ SSDE_5.25_ < 0.45	5	92.2%	3.6%	0.117
0.45 ≤SSDE_5.25_ < 0.5	14	93.0%	3.2%	0.013
0.5 ⩽ SSDE_5.25_	18	93.9%	3.4%	0.027

*Note*: For SAT 1.25 mm, tests for the smaller SSDE_5.25_ range showed that the lower limit of radiation dose required to achieve accuracy equal to or better than that due to differences in positioning was between 0.5 and 1.5 mGy for SSDE_5.25_.

Abbreviations: NRT, noise reduction techniques; SAT, subcutaneous adipose tissue; SSDE, size‐specific dose estimates.

**TABLE 5:** Using the manually calculated VAT value of the CT colonography (CTC) 5.25 mm image as ground truth, the accuracy of VAT with and without NRT for CTC images (5.25 and 1.25 mm), as well as the accuracy of SAT in each dose range with NRT, were tested against the average accuracy of VAT with positioning (85.2%).

	Samples	Mean	SD	*p*‐value
VAT manual (all days)	**45**	**85.2%**	**10.4%**	–
Without NRT				
VAT ‐ 5.25 mm	46	92.8%	9.8%	1.000
VAT ‐ 1.25 mm	46	85.3%	11.0%	0.534
With NRT				
VAT ‐ 5.25 mm (SSDE_5.25_ ≥ 1.5)	46	97.5%	2.7%	1.000
VAT ‐ 1.25 mm	46	94.0%	7.3%	1.000
0.35 ⩽ SSDE_5.25_ < 0.4	9	88.2%	13.4%	0.886
0.4 ⩽ SSDE_5.25_ < 0.45	5	98.3%	1.0%	1.000
0.45 ≤SSDE_5.25_ < 0.5	14	96.6%	3.2%	1.000
0.5 ⩽ SSDE_5.25_	18	93.8%	3.2%	0.999

*Note*: For VAT 1.25 mm, testing over the smaller SSDE_5.25_, the minimum dose required to achieve accuracy equal to or better than the accuracy of VAT measurements with different positioning was estimated to be SSDE_5.25_ ≥ 0.4 mGy.

Abbreviations: NRT, noise reduction techniques; SSDE, size‐specific dose estimates; VAT, visceral adipose tissue.

In the case of VAT, on the other hand, the 5.25 mm images were sufficiently accurate even without noise reduction (*p* = 1.0) (Table 5), showing no benefit from noise removal. However, for the 1.25 mm images, some data were not sufficiently accurate without noise reduction (*p* = 0.534), but noise reduction resulted in most of the data being above the acceptable level (*p* = 1.0). For each of the regions tested in SSDE_5.25_, the *p*‐value was almost 1 at 0.4 mGy and above, indicating that sufficient accuracy was obtained. Based on this result, the lower limit of SSDE_5.25_ required for VAT measurement is predicted to be about 0.4 mGy.

## 4 DISCUSSION

This study used a vendor‐agnostic, post‐processing NRT in combination with an automated fat quantification technique. Using this combination, we have effectively demonstrated that scans obtained at doses as low as those typically obtained for the purpose of CT colonography are acceptable for use with our automated fat quantification technique when a post‐processing NRT is employed. Furthermore, we have shown that this NRT theoretically facilitates accurate fat quantification on scans taken at doses as low as SSDE_5.25_ 1.5 mGy. However, the effectiveness and characteristics of NRTs provided by various vendors other than the technique used in this study are different, and each technique should be evaluated individually.

The effective dose required to perform SAT and VAT measurements was also studied. In the data used in this study, the mean scan length to cover T12 ‐ L5 was 16.8 cm (standard deviation 1.4 cm). Therefore, if a 16.8 cm scan is performed at 1.5 mGy, the effective dose would be about 0.38 mSv when calculated using 0.015 as the conversion coefficient for the abdomen and pelvis.^36^

Yamada et al. reported no effect of noise reduction on fat quantification in manual fat quantification,^21^ but in our study, we observed a significant improvement in fat quantification by noise reduction. The reason for this difference may be that in the case of manual fat quantification, the improvement in accuracy due to noise reduction was not observable due to the inter‐ and intra‐operator variability. Furthermore, the lower limit of the dose reported by Yamada et al. was approximately 2.3 mGy, but according to our results, 2.3 mGy may be too high to observe any improvement due to noise reduction.

A few limitations exist in this study. First, the dataset was influenced by the time between CTC and standard dose scans; a dataset at which the two scans were taken at the same time would be optimal. Second, a major limitation of this study is that it was performed on a single CT system with a single reconstruction kernel. A different CT system or different reconstruction kernel may have varying results, as well as different dose reduction methods.

## 5 CONCLUSIONS

By analyzing data with a slice thickness of 1.25 mm, we estimated that SSDE of 1.5 mGy or effective radiation dose of 0.38 mSv is the lower limit for measuring SAT and VAT on abdominal CT images with a slice thickness of 5.25 mm using the NRT we used and our automated fat quantification.

## AUTHOR CONTRIBUTIONS

Study design and fat quantification were done by Andrew T. Grainger. Application of the noise reduction technique and statistical analyses was done by Akira Hasegawa. The study was done under the supervision and guidance of Arun Krishnaraj.

## CONFLICT OF INTEREST STATEMENT

Andrew T. Grainger and Arun Krishnaraj report no conflicts of interest. Akira Hasegawa is an employee of AlgoMedica, Inc.

## INSTITUTIONAL REVIEW BOARD STATEMENT

This study was included in a retrospective research protocol approved by the IRB of the University of Virginia (Protocol # 17041). All images used in this study were de‐identified prior to processing, and all procedures were performed in compliance with the Health Insurance Portability and Accountability Act (HIPAA).
